# Electrochemical determination of zinc(II) using N^1^-hydroxy-N^1^,N^2^-diphenylbenzamidine and multi-walled carbon nanotubes modified carbon paste electrode

**DOI:** 10.1016/j.heliyon.2023.e17346

**Published:** 2023-06-15

**Authors:** Endale Tesfaye, Bhagwan Singh Chandravanshi, Negussie Negash, Merid Tessema

**Affiliations:** Department of Chemistry, College of Natural and Computational Sciences, Addis Ababa University, P.O. Box 1176, Addis Ababa, Ethiopia

**Keywords:** HDPBA-MWCNTs/CPE, Zn(II) determination, SWASV, Electrochemical behavior of HDPBA, Water samples

## Abstract

In this study, a new carbon paste electrode modified with a laboratory-synthesized ligand, N^1^-hydroxy-N^1^,N^2^-diphenylbenzamidine (HDPBA) and multi-walled carbon nanotubes (MWCNTs) (HDPBA‒MWCNTs/CPE) has been developed. The modified electrode was applied for preconcentration and voltammetric determination of zinc ions (Zn(II)) by square wave anodic stripping voltammetry (SWASV). The preconcentration of Zn(II) on the electrode surface was performed in 0.1 M Brinton Robinson (B–R) buffer solution (pH 6) at an applied potential of −1.30 V versus Ag/AgCl for 120 s, followed by stripping in the positive potential scan of the SWASV after a quit time of 10 s. Under optimized experimental conditions, the proposed electrode exhibited a wider linear dynamic response for Zn(II) in a concentration range of 0.02–10.00 μM with a detection limit of 2.48 nM. This is due to the excellent metal-chelation property of the ligand, and the good conductivity and large surface area of MWCNTs which significantly improved the sensing performance of the nanocomposite modified electrode. The selectivity of the electrode was studied by evaluating the interference effects of various foreign ions on the peak current of Zn(II). The method exhibited high reproducibility with a relative standard deviation (RSD) of 3.1%. The present method was applied for the determination of zinc ions in water samples. The recovery values in the tested samples were found to be 98.50–106.0%, indicating a good accuracy of the proposed electrode. Furthermore, the electrochemical behavior of HDPBA in acetonitrile and aqueous solutions has been studied.

## 1 Introduction

Due to their toxicity and non-biodegradable nature, heavy metal ions are recognized as a threat to human health and the environment [1]. Nowadays, because of the rapid expansion of industrialization, agricultural activities, and technology, the pollution of heavy metals (HMs) is rapidly growing in the environment. A high concentration of HMs will eventually reach and bioaccumulate in the human body through the food chain, which can pose a serious threat to human health [2]. It is clear that not all heavy metal ions are strictly harmful, and some are essential trace elements in the human body and biological systems. However, these essential metal ions have an optimum concentration limit in the human body, and their excess or insufficient content will lead to various diseases [3]. Thus, because of the serious risk effects of HMs on human health and their harmfulness to the environment, monitoring the concentration of toxic heavy metal ions in the environment is highly important to improve the quality of the environment and human health. HMs contamination in the water and food chains may occur through different sources and routes. Water gets polluted by heavy metal ions and other ions through release from mining and manufacturing, disposal of metal wastes, rock weathering, fertilizer, pesticide use, etc. [4,5,6]. Therefore, the determination of trace metals in water is highly valuable to ensure and control its quality.

Zinc has significant benefits for human health, growth, and the maintenance of the human body at a trace level. It plays critical roles in human physiological activities and life processes, including the function of metalloenzymes, synaptic neurotransmission, gene expression, neuronal excitotoxicity, etc. [7,8]. However, zinc ions can accumulate in the human body when ingested in excess amounts beyond recommended levels. An excess concentration of zinc ions in drinking water and food causes several health problems, such as stomach pain, abdominal pain, vomiting, nausea, diarrhea, lethargy, depression, lethargy, and a short-term metal-fume fever. In severe cases, shortness of breath, high blood pressure, dilated pupils, convulsions, and shock are some of the health risks from the consumption of water and food contaminated with excess zinc ions [9,10]. On the other hand, the lack of zinc ion can cause many health problems, including immunity depression, growth retardation in children, and a detrimental impact on neuronal development, eye and skin dysfunctions, and organ diseases (skeleton system, gastrointestinal tract, etc.) [7,11]. Moreover, a lack of zinc ion concentration in the human brain causes diseases like epilepsy, Parkinson's, and Alzheimer diseases [12].

The detection of Zn(II) in various types of environmental, food, and biological samples is very important due to the nutritional and toxic effects of the metal ion or its compounds. The limit recommended by the World Health Organization (WHO) for Zn(II) in drinking water is 3 mg/L. Thus, the determination of Zn(II) in environmental water and drinking water samples becomes important to control and maintain their quality and to avoid excess intake or deficiency. Consequently, the development of an accurate, cost-effective, rapid, friendly-user, highly sensitive and selective method for the determination of trace Zn(II) is required.

Different types of analytical techniques have been used for the determination of Zn(II) and other HMs with high sensitivity [13,14,15,16]. However, these techniques suffer from limitations such as being time-consuming, requiring special preparation of samples and matrix matching, having high instrument and maintenance costs, requiring skilled personnel to operate, and not being suitable for an on-site determination [17]. To solve these limitations, electrochemical methods, particularly stripping voltammetric techniques have been widely used for the determination of metal ions, including Zn(II) due to their low-cost instrumentation, easy operation, ability to detect multi-elements, excellent detection limits, high sensitivity, and high stability [18,19]. Among various voltammetric techniques, square wave anodic stripping voltammetry (SWASV) is the most effective technique for the trace level detection of ionic species due to its additional advantages such as the ability to preconcentrate the target analyte, rapidity, simplicity in instrumentation, low electrical power consumption, and suitability for computerization. Since excellent sensitivity of the SWASV technique is primarily achieved by a preconcentration (accumulation) step in which target species are accumulated and reduced on the electrode surface, the behaviors of the electrode materials are significantly important [20]. Hence, the development of suitable electrodes for effective electrochemical sensing of HMs is crucial.

Among different types of electrodes, carbon paste electrodes (CPEs) have been commonly applied in the electrochemical sensing of several ions, including heavy metal ions. CPEs have several benefits, such as being simple to use, having excellent reproducibility, and being able to modify easily. Carbon-based materials are mostly used for the fabrication of paste electrodes due to their many advantages, including the ability to bind with other substances, form a homogeneous electrode, and have good conductivity. Furthermore, the advantages of carbon pastes include their environmental friendliness, non-toxicity, inexpensive preparation, large electrochemical potential windows, low background current, etc. [21,22]. Modified carbon paste electrodes (MCPEs) are more efficient and suitable tools for the detection of heavy metals and have been widely used recently due to their regenerating ability, simple preparation, large surface area, and high selectivity and sensitivity [23].

**Fig. 1:**
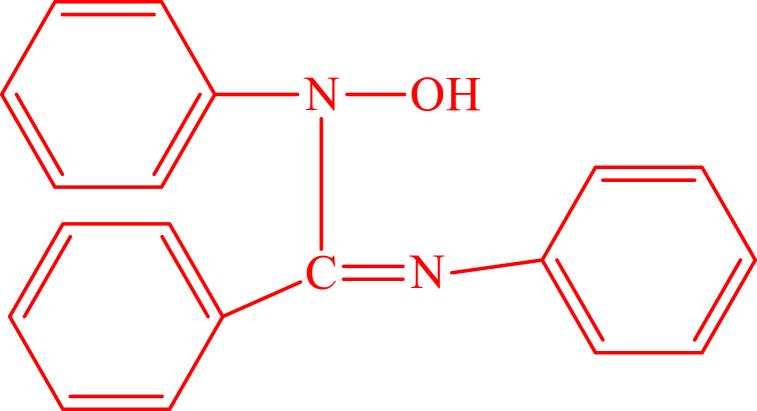
Structure of N^1^-hydroxy-N^1^,N^2^-diphenylbenzamidine (HDPBA).

CPEs modified with metal chelating ligands have been widely used for the detection of metal ions because of their chelating capability to target heavy metal ion selectively. The incorporation of water-insoluble ligands into the carbon paste electrode matrix can be exploited for further formation, adsorptive accumulation, and reduction of a surface active complex of the metal. Most adsorptive analyses with ligand modification involve the reduction of the metal in the adsorbed complex at the electrode surface. It is also possible to exploit the redox behavior of the ligands. This is especially helpful for the metal ions, which do not show distinct redox peaks while the ligands are electrochemically active and give well-quantified peaks in the potential range used [24]. N^1^-Hydroxy-N^1^,N^2^-diphenylbenzamidine (HDPBA) (Fig. 1) is a typical monobasic and bidentate chelating agent and has the capability to form stable complexes with target metal ions. The electron donor atoms like O and N in the HDPBA chemical structure have offered the ligand a high tendency to form relatively stable metal complexes with metal ions, making it a suitable modifier in the development of metal ion electrodes. To the best of our knowledge, the complex formation reaction and voltammetric behavior of Zn(II) by using the HDPBA modified carbon paste electrode have not been investigated.

Furthermore, special characteristics such as large surface area, high conductivity, and high mechanical strength make the multi-walled carbon nanotubes (MWCNTs) unique materials, and they have been widely used in the fabrication of electrochemical electrodes [25,26,27]. The use of MWCNTs with metal chelating ligands (like HDPBA) nanocomposites as modifiers of carbon paste electrodes can provide better sensing performance to the electrode for the detection of metal ions due to the cumulative advantages such as high surface area, good adsorptive capability, high conductivity of the MWCNTs, and an excellent chelating capability of the specific ligands [28]. The selectivity and sensitivity of the determination of the metal ion by adsorptive stripping voltammetry can be improved by its interactions with various functional groups of the ligand and the functionalized multiwalled carbon nanotubes. The combination of such properties in the nanocomposite can result in high sensitivity and selectivity of the modified electrode. This finally results in low detection limits and a wide linear range in the voltammetric determination of a target metal ion [25,26,27,28].

Therefore, the voltammetric determination of Zn(II) at ligand (HDPBA) and MWCNTs composite modified CPE (HDPBA‒MWCNTs/CPE) was studied in the present investigation. In addition, the electrochemical behavior of HDPBA has not been investigated so far. Hence, the voltammetric behavior of HDPBA in acetonitrile and aqueous/buffer solutions using SWASV and cyclic voltammetry (CV) has also been investigated in this study.

## 2 Experimental

### 2.1 Apparatus and chemicals

A PerkinElmer UV–Vis spectrometer (Lambda 950, USA) was used for the recording of the UV–Vis spectra. An electrochemical analyzer (CHI 840C, USA) was applied for the voltammetric measurements throughout the study. A personal computer (IBM, 130100DX4), which was connected to the electrochemical analyzer, was used for data processing and recording voltammograms. A three-electrode system with a reference electrode (Ag/AgCl), a working electrode (unmodified or modified carbon paste electrode), and an auxiliary electrode (platinum wire) was employed in all the voltammetric measurements. The atomic absorption spectrometer (AAS, Agilent 280Z AA Zeeman) was used for atomic absorption spectroscopic (AAS) analysis of real samples.

Multi-walled carbon nanotubes (MWCNTs, 99.8%, Sigma-Aldrich), paraffin oil (density at 25 °C, 0.845−0.905, Carlo Erba Reagents), and carbon powder (99.5%, BDH Laboratory Supplies, Poole) were used for the preparation of carbon paste. Different concentrations of Zn(II) solutions were prepared from a 1000 mg/L standard solution of Zn(II) throughout the study. Brinton Robinson (B–R) buffer was used for the preparation of the supporting electrolyte solution. Britton Robinson (B–R) buffer solution was prepared using acetic acid (99.8%, Sigma-Aldrich, USA), phosphoric acid (85.0%, Riedel-de Haen Chemicals, Germany), and boric acid (99.5%, Carlo Erba Reagents, Cornaredo, Italy). The pH of the B–R solution was adjusted to suitable values by adding sodium hydroxide solution (98.0%, Avonchem, United Kingdom). Other chemicals such as ammonium chloride (99.5%, Merck), sodium phosphate (99.0%, BDH Chemicals Ltd., England), sodium acetate (99.0%, BDH Chemicals Ltd., England), ammonia (35%), hydrochloric acid (37% Riedel-de Haen Chemicals, Germany), citric acid (99.0%, Research-lab Fine Chem Industries, Mumbai), and sodium citrate (98.0%, Research-lab Fine Chem Industries, Mumbai) were used for the preparation of several solutions in the selection of a suitable buffer solution. Salts of other metal ions (such as sulfate, nitrate, and chloride salts) were used in the interference study. Functionalization of MWCNTs was performed using nitric acid (69.5%, Scharlau) and sulfuric acid (97%, Scharlau). HDPBA was prepared by the condensation reaction of N-phenyl- benzimidoyl chloride with N-phenylhydroxylamine in ether at 0 °C [29].

Lithium perchlorate (LiClO_4_, 99.0%), tetrabutylammoniumhexafluorophosphate (TBAHFP, 98.0%, Sigma-Aldrich), and acetonitrile (99.8%, Carlo Erba Reagents, Cornaredo, Italy) were used as solvents in the electrochemical behavior study of the HDPBA at the glassy carbon electrode (GCE). While acetate buffer solution (ABS) was used as a supporting electrolyte solution during the electrochemical behavior study of HDPBA (7.5%) modified carbon paste electrode (HDPBA‒CPE). Solutions of TBAHFP (10 mM), LiClO_4_ (0.1 M), and HDPBA (0.1, 0.5, 1.0, and 2.0 mM) were prepared in acetonitrile. Acetate buffer solutions (sodium acetate, 0.3 M) of pH 3–12 were prepared in distilled water. The pH of the ABS solution was adjusted by adding acetic acid and 1 M sodium hydroxide [30].

### 2.2 Pretreatment of MWCNTs and preparation of electrodes

In order to avoid impurities and amorphous carbon, pretreatment and functionalization of MWCNTs were performed using sulfuric and nitric acid (3:1 (v/v)) for 12 h at room temperature. The suspension was then filtered, and the residue was washed with distilled water. Finally, drying of the washed solid at 120 °C for 5 h was performed [31].

The unmodified CPE was prepared by mixing carbon powder (1.00 g) with paraffin oil (0.36 mL) in a pestle and mortar for 25 min. A portion of the mixture was then packed into the tip of the syringe (5 cm in length and 3 mm in outer diameter) with a copper wire for electric contact. Then polishing of the prepared electrode surface was performed on clean paper until a smooth and shiny surface was obtained. The modified carbon paste electrodes were made in a similar manner by substituting the corresponding contents of the graphite powder with MWCNTs and HDPBA in different ratios. A fresh surface was obtained by removing a portion of the used surface and substituting it with fresh paste whenever renewal of the electrode was needed. The fresh electrode was pretreated by immersing it in a supporting electrolyte solution (B–R buffer, 0.1 M) and recording the voltammograms 3–5 times to obtain reproducible results.

### 2.3 Analytical procedures

The determination of Zn(II) using the prepared electrode was performed by applying accumulation and stripping steps. First, a solution of Zn(II) with a known concentration was added into the cell containing B–R buffer solution (0.1 M, pH 6). The accumulation of Zn(II) at the surface of the electrode was then performed after immersing the electrode in the solution by applying a potential of −1.30 V for 120 s while stirring the solution. The stirring of the solution was then ceased, and 10 s of quit time were allowed in order to settle the solution. Finally, the voltammograms were recorded in the potential range of −1.7 V to −0.50 V (positive scan) versus Ag/AgCl. The electrode was renewed and pretreated at the end of each voltammetric measurement using 3–5 runs/scans in 0.1 M B–R buffer solution (pH 6).

The electrochemical behavior study of the ligand (HDPBA) was performed using acetonitrile/LiClO_4_/TBAHFP solutions (at a glassy carbon electrode (GCE)) and ABS (at a carbon paste electrode, a 7.5% HDPBA-modified CPE). Various buffer solutions such as acetate buffer solution (ABS), ammonium chloride, Brinton Robinson (B–R) buffer, and phosphate buffer solution (PBS) were tested to select the best solution for the electrochemical behavior study of the ligand in aqueous solutions. The dependence of the peak potential and current of HDPBA on the pH of the solution was performed in 0.3 M sodium acetate solution in the pH range of 3–12 using square wave anodic stripping voltammetry (SWASV).

### 2.4 Real samples analysis

To investigate the suitability of the prepared electrode for real sample analysis, tap water and industrial wastewater samples were collected from Addis Ababa University (Science Campus) and Akaki River, respectively. After collection, the water samples were filtered through a Whatman filter paper in order to remove solid particles. Finally, their pHs were adjusted to the suitable values, and the quantification of Zn(II) in the water samples was done by applying the standard addition method. The proposed method was validated by determining Zn(II) in the water samples using the atomic absorption spectroscopic (AAS) technique.

## 3 Results and discussion

### 3.1 UV–Vis absorption spectra of HDPBA and its complexation formation with metal ion

**Fig. 2:**
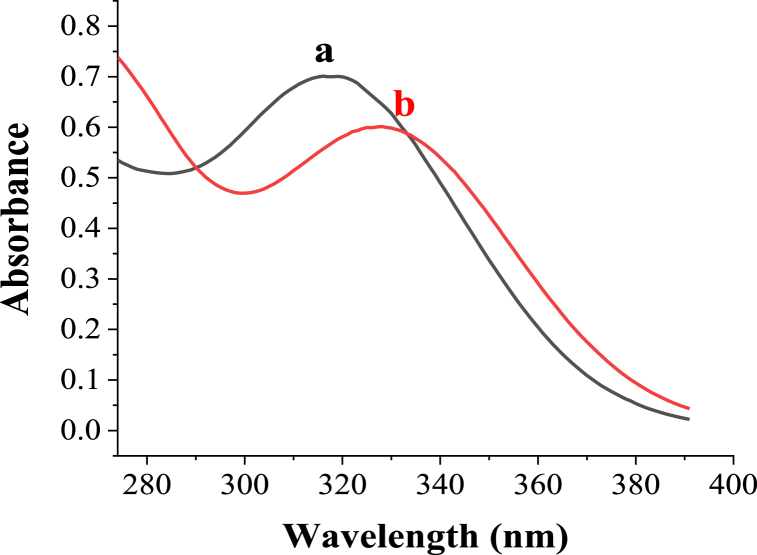
UV–Vis absorption spectra of HDPBA (6 × 10^−5^ M) (**a**) without the addition of Zn(II) and (**b**) with the addition of Zn(II) in ethanol.

The interaction of Zn(II) with the ligand (HDPBA) was studied using UV–Vis spectrometry in ethanol solution. The UV–Vis spectra for ligand (HDPBA) before and after the addition of Zn(II) are shown in Fig. 2. As can be seen in the figure, the ligand (HDPBA) without the addition of Zn(II) has an absorption spectrum with *λ*_max (ethanol)_ at 317 nm which is attributed to the n-π* transition of the C=N functional group in HDPBA (spectrum a). However, significant peak change/shift in the spectrum of HDPBA is found with the addition of Zn(II) (spectrum b). This spectrum shift with the addition of the metal ion to the ligand proves the complex formation between the Zn(II) and HDPBA. The complex formation between the Zn(II) and HDPBA results in bathochromic shift of n-π* transition of the C=N functional group in HDPBA. Similar shifts of n-π* transition absorption band of other ligands upon complex formation with other metals have also been reported [32,33,34,35].

### 3.2 Voltammetric determinations of Zn(II) with prepared electrodes

#### 3.2.1 Square wave anodic stripping voltammetric determination of Zn(II)

The unmodified and modified CPEs were evaluated for the determination of Zn(II) by square wave anodic stripping voltammetric measurements. Voltammetric determinations of Zn(II) using the electrodes were conducted after electrochemical accumulation of Zn(II) in buffer solution with an applied accumulation potential of −1.30 V for 120 s followed by potential scanning/stripping from −1.7 V to −0.50 V under square wave voltammetry conditions.

**Fig. 3:**
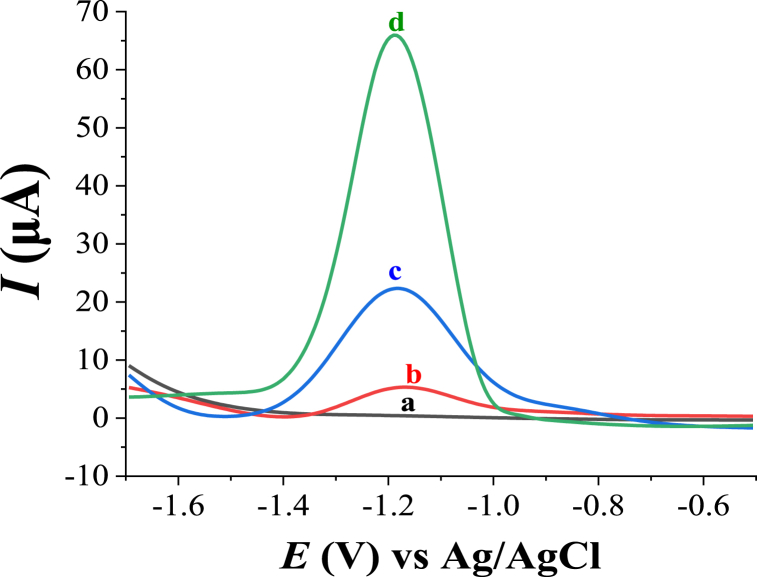
Square wave anodic stripping voltammograms (SWASVs) of 0.1 M buffer solution (B–R, pH 6) at HDPBA‒MWCNTs/CPE without the accumulation of Zn(II) (**a**), and with an accumulation of 2.0 μM Zn(II) at unmodified CPE (**b**), MWCNTS/CPE (**c**), and HDPBA‒MWCNTs/CPE (**d**); accumulation time: 120 s; accumulation potential −1.30 V; pulse amplitude: 60 mV; frequency: 40 Hz; and step potential: 6 mV.

Fig. 3 depicts square wave voltammograms of Zn(II) found using unmodified and modified electrodes. In the absence of Zn(II), no anodic peaks of Zn(II) were observed for unmodified CPE (not shown) and modified electrode, HDPABA‒MWCNTs/CPE (peak a) in B–R buffer solution (0.1 M, pH 6). However, in the presence of Zn(II), unmodified CPE exhibits a small anodic peak of Zn(II) at −1.165 V (peak b), which is assigned to the oxidation of zinc (Zn^0^ → Zn^2+^ + 2e^‒^). While a higher anodic peak at −1.190 V was observed for MWCNTs modified carbon paste electrode (MWCNTs/CPE) (peak c) compared to the unmodified electrode. This might be because of MWCNTs facilitate the charge transfer rate among metal ions and the electrode surface and enhances the accumulation of metal ions on the electrode surface due to good conductivity and the large surface area of the carbon nanotubes.

When MWCNTs/CPE was further modified with HDPBA (HDPBA‒MWCNTs/CPE) the peak signal of Zn(II) was further increased (peak d), this indicates HDPBA‒MWCNTs composite effectively enhances the voltammetric signals of Zn(II). This significant enhancement in the anodic peak current response of Zn(II) using HDPABA‒MWCNTs/CPE can be ascribed to the ligand (HDPBA) for good accumulation of Zn(II) on the electrode surface through the complexation reaction. The functional groups of prepared ligand (HDPBA) behave as a Lewis base and coordinate with the metal ions using the lone pair electrons on oxygen and nitrogen atoms having comparatively low π-acidity. Furthermore, the functionalization of the MWCNTs was conducted to remove impurities such as metallic compounds and carbonaceous materials and to introduce carboxylate and carboxyl functional groups at the ends and/or at the sidewall of the carbon nanotube structure that can enhance the conductivity and charge transfer rate of the electrode; this leads to increase in the sensitivity of the voltammetric responses of Zn(II) at HDPABA‒MWCNTs/CPE [31,36]. Therefore, the use of HDPABA‒MWCNTs composite for the modification of carbon paste electrode results in highly sensitive and selective determination of Zn(II).

#### 3.2.2 Proposed mechanism for the detection of Zn(II)

From the results obtained in the present study and similar research works found in the literature on the detection of metal ions with ligand modified electrodes, a mechanism for binding of Zn(II) at the electrode surface is proposed [37,38]. HDPBA contains nitrogen and oxygen functional groups which have active sites to form complexes with the Zn(II). The mechanism for the preconcentration and oxidation of Zn(II) at the surface of the modified electrode is given as follows (where HL represents HDPBA):

Accumulation/reduction step:

(Zn^2+^) _solution_ + (2HL) _CPE surface_ → (ZnL_2_) _CPE surface_ + (2H^+^) _solution_

(ZnL_2_) _CPE surface_ + (2H^+^) _solution_ + (2e^−^) _CPE surface_ → (Zn) _CPE surface_ + (2HL) _CPE surface_

Stripping step:

(Zn) _CPE surface_ → (Zn^2+^) _solution_ + 2e^-^

The schematic representation of the proposed structure of the complex between the Zn^2+^ and HDPBA is shown in Fig. S1.

### 3.3 Optimization studies

#### 3.3.1 Effect of the electrode compositions

**Fig. 4:**
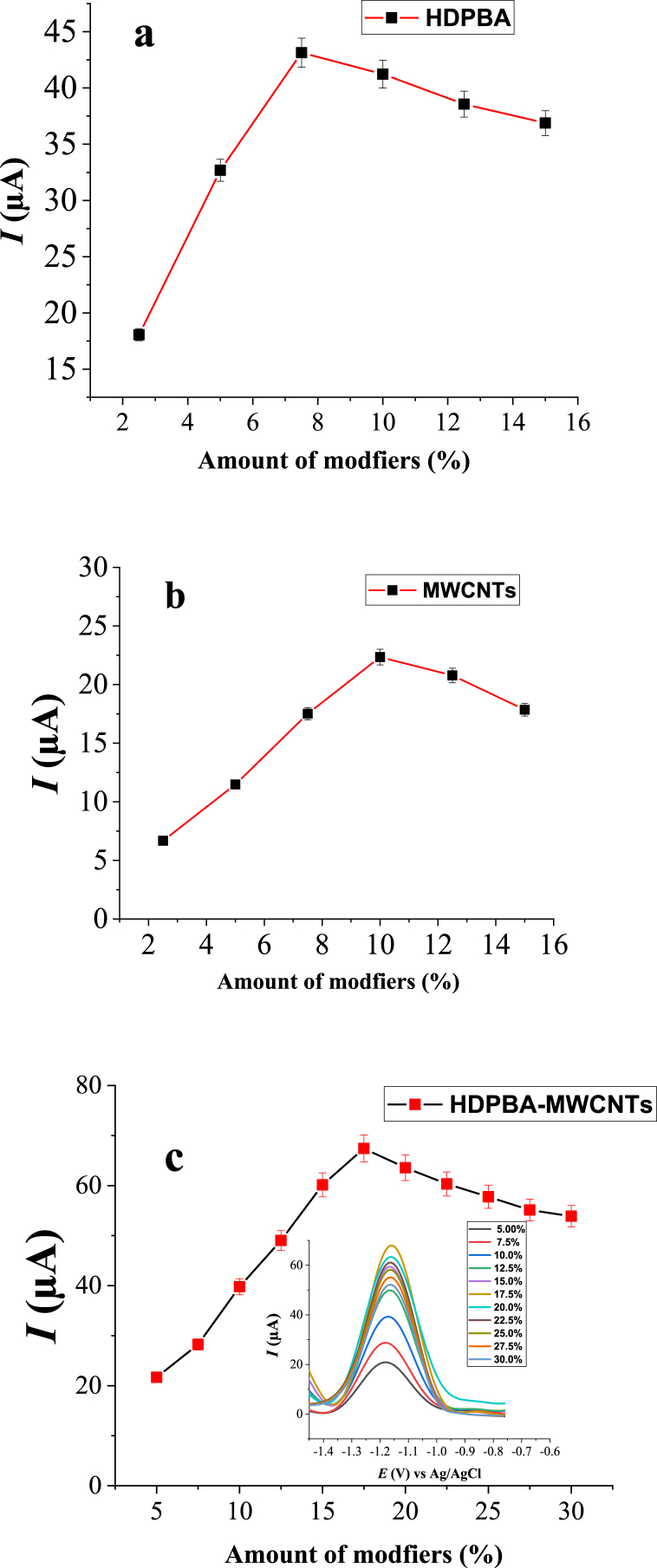
Effect of modifiers content ((**a**) MWCNTs, (**b**) HDPBA, and (**c**) HDPBA‒MWCNTs) on the peak current of 2.0 μM Zn(II) in buffer solution (B–R, 0.1 M). Other experimental and instrumental parameters are as in Fig. 3.

The effect of composition of electrode on the peak current of Zn(II) was studied using the modified CPE prepared with different contents of the modifiers (HDPBA and MWCNTs) from 2.5% to 15% (w/w) for each modifier. As shown in Fig. 4a, at a constant composition of MWCNTs (10% (w/w)), an increase in the current response of Zn(II) was obtained with increasing the amounts of HDPBA from 2.5% to 7.5%. This is ascribed to the presence of a higher content of accumulation/adsorption sites coming from the HDPBA, which results in the formation of higher amounts of Zn(II) complex on the surface of the electrode and consequently to a higher peak current of Zn(II). However, CPEs prepared using HDPBA amounts higher than 7.5% (w/w) have exhibited a decrease of peak current response which is ascribed to the small content of conductive graphite in the carbon paste composition which leads to a decrease in the conductivity of the electrode, since the ligand (HDPBA) is a non-conductive material. In the case of the MWCNTs content, by keeping the amount of HDPBA constant (7.5%), the peak current was increased with the increasing amount of MWCNTs from 2.5% to 10% (w/w) (Fig. 4b). This is because of the excellent conductivity and high surface area of the MWCNTs. However, no significant peak signal enhancement was observed after 10.0% (w/w) of MWCNTS, this may be because of the nanomaterials agglomeration effect at higher contents. Therefore, 82.5% of carbon paste (graphite powder plus paraffin oil), 7.5% of HDPBA, and 10.0% of MWCNTs (Fig. 4c) were used as an optimum electrode composition for further studies.

#### 3.3.2 Effect of buffer solutions and pH

**Fig. 5:**
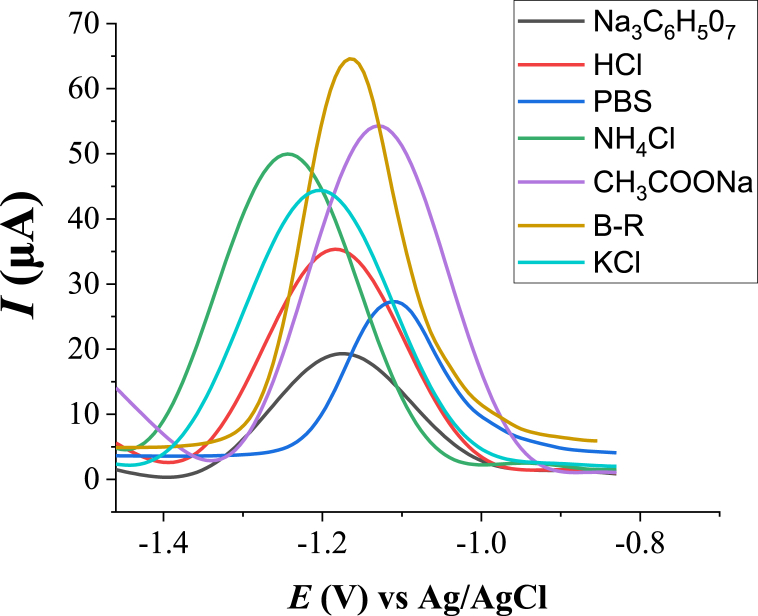
Effects of various supporting electrolytes on the peak current response of 2.0 μM Zn(II). Other experimental and instrumental parameters are as in Fig. 3.

Various buffer solutions including acetate buffer (sodium acetate), ammonium chloride, Brinton Robinson (B–R) buffer, hydrochloric acid (HCl), sodium citrate, phosphate buffer solution (PBS) and potassium chloride (KCl) were tested to select the best supporting electrolyte solution for the determination of Zn(II) at the HDPBA‒MWCNTs/CPE using SWASV. As can be seen in Fig. 5, the peak current obtained from a Brinton Robinson buffer (0.1 M) was relatively higher than other buffer solutions.

**Fig. 6:**
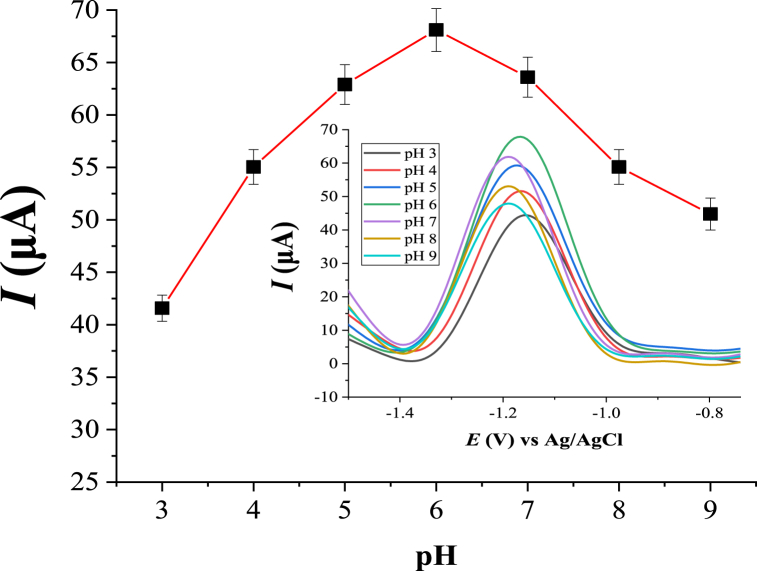
Effect of pH of buffer solution (B–R, 0.1 M) on the peak current of 2.0 μM Zn(II). Other experimental and instrumental parameters are as in Fig. 3.

The B–R buffer solution pH values in the range of 3.0 to 9.0 were tested in order to investigate the effect of pH on the determination of Zn(II). The dependence of the peak signals with a variation of pH is depicted in Fig. 6. An increase in the peak current of Zn(II) with increasing pH was obtained up to pH 6.0. At lower values of pHs (<6.0), the peak response of Zn(II) was decreased. This might be attributed to the competition between Zn(II) and the proton for ligand (HDPBA) sites and/or the reduction process. On the other hand, pH values after 6.0, the peak intensities were decreased. The decrease of the anodic peak current at higher pHs of buffer solution (pHs *>* 6) may be due to the hydrolysis of the Zn(II) resulting in decreased complex formation with the ligand. Based on the obtained results, the B–R buffer solution of (pH 6.0, 0.1 M) was chosen as the optimum pH to be used in subsequent experiments. There is a slight change in peak potential with pH changes. This may be because of the involvement of proton (H^+^) in the reaction. Since the ligand (HDPBA) has –OH functional group on its structure, there would be protonation/deprotonation process during the reaction/complex formation between the ligand and the metal ion, Zn(II). This protonation/deprotonation process leads a change in peak potentials with pH change. Furthermore, at extremely lower pH, there would be decomposition of the complex due to protonation of the ligand and hence it loses its ability for complex formation with the metal ion. This means the proton will compete with Zn(II) for binding to the donating atoms of the ligand at the surface of the electrode at very lower pH.

#### 3.3.3 Accumulation time and potential

**Fig. 7:**
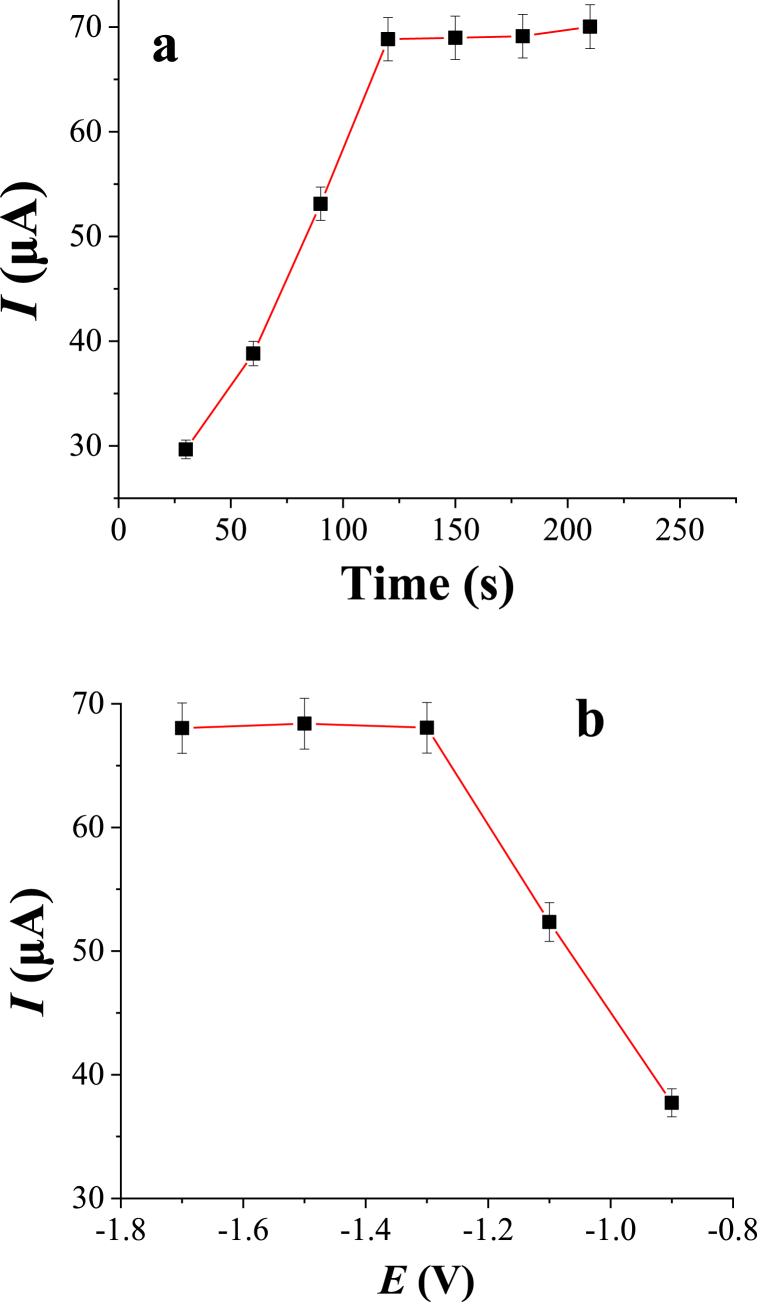
Effect of accumulation time (**a**) and accumulation potential (**b**) on the peak current of 2.0 μM Zn(II). Other experimental and instrumental parameters are as in Fig. 3.

In order to investigate the effect of the accumulation time on the current response of Zn(II), the measurements were conducted by varying the accumulation times from 30 to 210 s under previously optimized parameters. The results are shown in Fig. 7a. As can be seen in the figure, an increase in peak intensities of Zn(II) was found with increasing accumulation time up to 120 s. However, the observed increment was not continued, peak currents of Zn(II) were increased slightly above 120 s. This is probably attributed to the saturation of accumulation/adsorption sites present at the electrode surface, which makes the slight increment of the peak intensity. Hence 120 s was selected as an optimum accumulation time.

The dependence of stripping peak currents of Zn(II) on the accumulation potential was conducted by changing the potential values in the range between −0.90 V and −1.70 V versus Ag/AgCl. As can be seen in Fig. 7b, the peak current of Zn(II) significantly enhanced with the negative potential values up to −1.30 V, and the larger current response was found at −1.30 V. The lower peak currents at more positive potentials (<−1.30 V) is due to insufficient reduction of target metal ion (Zn(II)) because of inadequate reduction potential. A slight decrement in the peak current responses was observed after a potential of −1.30 V. This might be due to the formation of hydrogen evolution/reduction of hydrogen ions in the test solution when more negative potential (>−1.30 V) was applied on the electrode. Therefore, an accumulation potential of −1.30 V was used for subsequent measurements.

#### 3.3.4 Effect of scan rate (v)

**Fig. 8:**
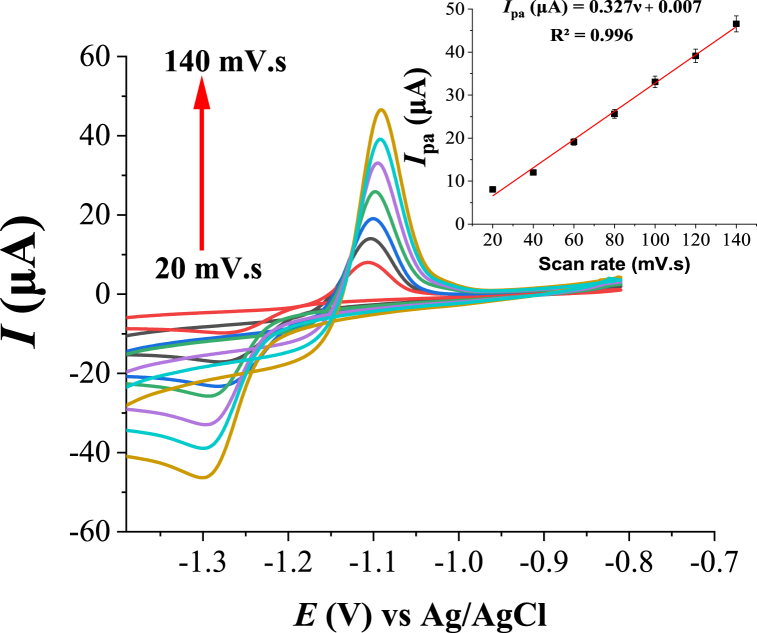
Cyclic voltammograms of 2.0 μM Zn(II) in 0.1 M buffer solution (B–R, pH 6) at HDPBA‒MWCNTs/CPE with different scan rates (bottom to top: 20, 40, 60, 80, 100, 120 and 140 mV s^−1^). Inset: Effect of variation of scan rate on the anodic peak current of Zn(II).

To investigate the type of electrochemical mechanism occurred between the Zn(II) and the modified electrode, the effect of scan rate (*v*) on the peak current (*I*_p_) and peak potential (*E*_p_) was investigated using cyclic voltammetry. As shown in Fig. 8, the anodic peak current (*I*_pa_) linearly increases with the increase in the scan rate from 20 to 140 mV s^−1^. Furthermore, from the plot of log I_pa_ versus log *v*, the slope of the linear equation was found to be 0.929. This indicates that the electrochemical mechanism involves a surface adsorption process. The relationship between the scan rate and the peak potential (*E*_p_) was also studied. If the electro-oxidation reaction is irreversible, *E*_p_ is dependent on *v*, and vice versa. From Fig. 8, the anodic peak potential (*E*_p_) shifts to a higher value with the increase in the scan rate indicating the dependence of *E*_p_ on *v*. This suggested that the electron transfer in analyte electro-oxidation is irreversible.

#### 3.3.5 Effect of instrumental conditions

**Fig. 9:**
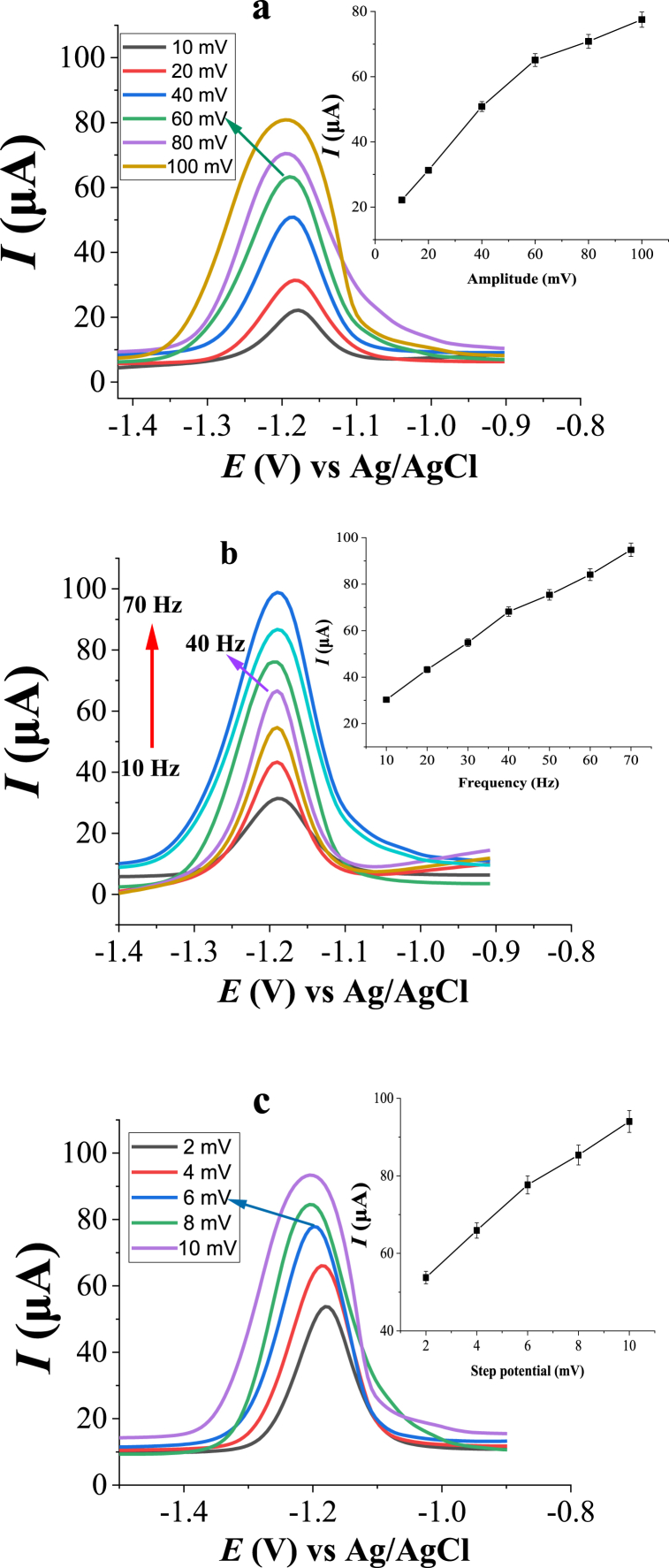
Effect of instrumental conditions (**a**) pulse amplitude, (**b**) frequency, and (**c**) step potential increment on the peak current of 2.0 μM Zn(II). Other experimental and instrumental parameters are as in Fig. 3.

The instrumental setup conditions including potential pulse amplitude, frequency, and potential increment have significant effects on the peak currents of the metal ion. The influence of amplitude on the peak response of Zn(II) was investigated by changing the amplitude values from 10 to 100 mV. The peak current enhanced and became broader as the amplitude increased from 10 to 100 mV (Fig. 9a). To compromise between the peak height and peak width, 60 mV was selected as the optimum amplitude and used for subsequent measurements. The effect of frequency was determined by varying its values from 10 to 70 Hz. Based on observed results and considering the symmetry and the peak intensity, 40 Hz was selected as the optimum value (Fig. 9b). The influence of step potential increment on the peak current was studied by changing the values from 2 mV to 10 mV. Based on the obtained results a step potential increment of 6 mV was chosen and used for further studies (Fig. 9c).

### 3.4 Analytical performance of the developed electrode

**Fig. 10:**
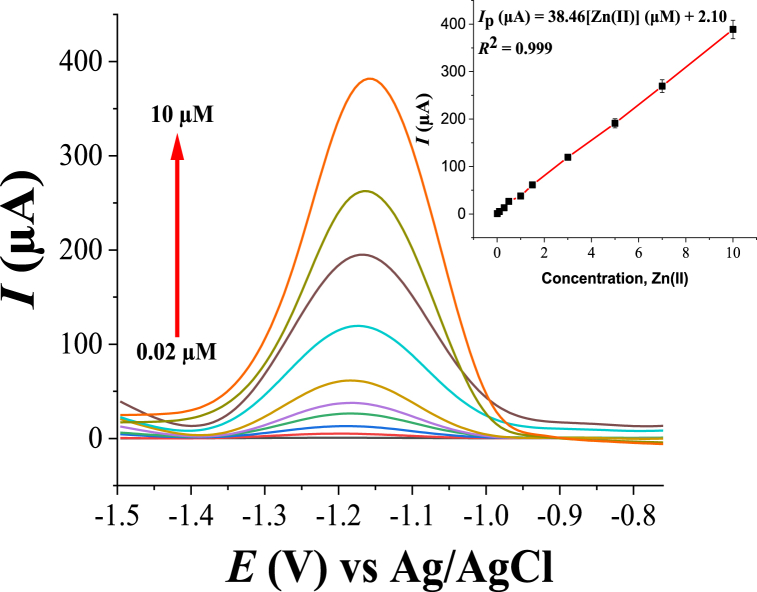
SWASVs of various concentrations (bottom to top: 0.02, 0.10, 0.30, 0.50, 1.00, 1.50, 3.00, 5.00, 7.00, and 10.00 μM) of Zn(II). Inset: the corresponding Zn(II) concentrations versus peak currents. Other experimental and instrumental parameters are as in Fig. 3.

After the selection of the optimum experimental and instrumental conditions for the determination of Zn(II) using SWASV, calibration plots were made based on triplicate measurements of different concentrations of Zn(II). The voltammograms and the corresponding calibration curves are shown in Fig. 10. From the calibration curves, a linear range of 0.02–10 μM of Zn(II) with a correlation value (R^2^) of 0.999 was obtained. The limits of detection (LOD, as calculated by 3.3*S/m (where S is the standard deviation of signals of 0.02 μM Zn(II) and m is slope of the calibration curve) and the limit of quantification (LOQ, ten times the standard deviation of signals of 0.02 μM Zn(II)/calibration curve slope) were found to be 2.48 nM and 7.46 nM, respectively. The standard deviation was calculated by estimation of six repeated determinations of 0.02 μM of Zn(II) signals. According to these obtained results, it can be concluded that the proposed HDPBA‒MWCNTs/CPE has excellent analytical performance for Zn(II) determination at the trace levels.

Furthermore, the analytical performance of the developed modified electrode was compared to the previously reported electrodes for zinc ions determination. The comparison of the analytical characteristics of several previously reported modified electrodes and the developed sensor for the determination of Zn(II) are given in Table 1. As can be seen from the table, the proposed electrode exhibited a very low detection limit and wider linear dynamic range which are better than most of the reported electrodes in the literature. The present electrode has also a shorter and comparable accumulation time with most of the reported electrodes. Furthermore the anodic peak potential of Zn(II) at the proposed electrode, and the reproducibility and sensitivity of the electrode were compared with other reported modified electrodes in Table 1. From the table, the proposed electrode exhibited lower RSD value [40,41,43] and higher slope than other electrodes which indicates the better reproducibility and sensitivity of the present electrode as compared to the reported electrodes. The peak potential of Zn(II) at the proposed electrode was found to be −1.19 V which is in good agreement with other reported values.

**Table 1:** Comparison of the analytical performance of HDPBA‒MWCNTs/CPE with other electrodes reported in the literature for the detection of Zn(II).

Electrode^a^	Technique	Preconcentra-tion time (s)	Liner range (nM)	LOD (nM)	Reproducibility, RSD (%)	Sensitivity (μA/μΜ)	E_pa_ (V)	Ref.
Hg nanodroplets Supported at biochar-modified CPE	DPASV	180	500–30000	170	–	–	‒1.10	[39]
Boron-doped diamond Electrode	DPV	240	76.50–624.3	2.45	<5	14.1	≈ −1.20	[40]
MCPE modified with chelating resin	DPV	90	153–765	109	4.0	38.3	‒1.05	[41]
Cr‒CPE	SWASV	100	1224–12240	382	–	0.71	≈ −1.20	[42]
Bismuth film-modified Electrode	DPASV	110	76.50–1683	16.7	1.74	–	≈ −1.18	[10]
G/PANI/SPE	SWASV	–	15.30–4590	15.3	9.2	1.48	‒1.31	[43]
Hg–Bi/SWNTs/GCE	SWASV	120	7.650–1989	3.57	–	–	‒1.08	[44]
HDPBA‒MWCNTs/CPE	SWASV	120	20–10000	2.48	3.1	38.5	‒1.19	Present work

a MCPE: modified carbon paste electrode; Cr: chromium(III) oxide; G/PANI: graphene polyaniline nanocomposite; Hg–Bi/SWNTs: Bimetallic Hg–Bi/single-walled carbon nanotubes composite.

The reproducibility of the proposed electrode was investigated by the detection of 0.5 μM Zn(II) solution using 10 separate electrodes made in the same way. A relative standard deviation (RSD) of 3.1% was found for the 10 separate electrodes, which indicates the good reproducibility of the fabricated electrode. The repeatability of HDPBA‒MWCNTs/CPE was investigated by conducting ten repeated determinations of 0.5 μM Zn(II) solution with the same electrode. The RSD value for ten repeated determinations of 0.5 μM Zn(II) solution was found to be 2.0%. The stability of the proposed modified electrode was evaluated by comparing the voltammetric peak current responses of 1.0 μM Zn(II) over two months. The relative error in the peak current signal after two months was found to be 5.9%. The results proved that the prepared modified electrode has good reproducibility and stability.

### 3.5 Interferences study

**Fig. 11:**
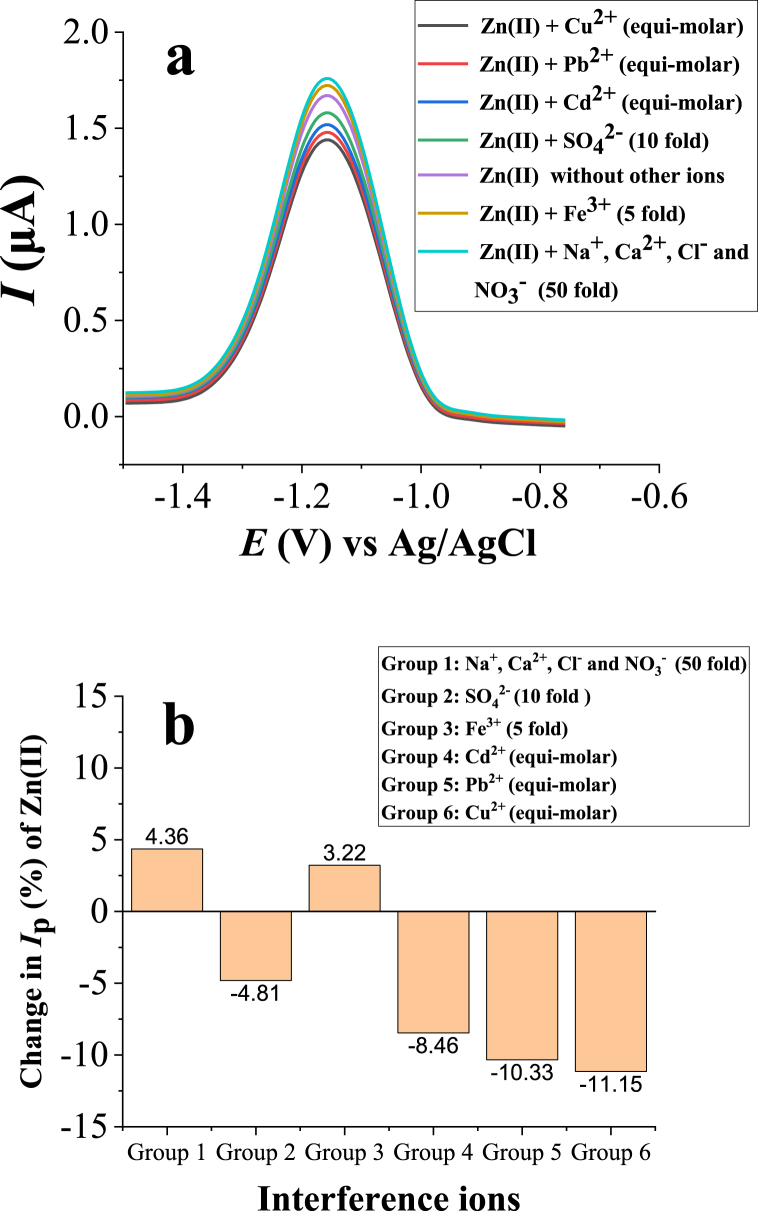
SWASVs of 0.05 μM Zn(II) in absence and presence of other ions with HDPBA‒MWCNTs/CPE (**a**) and the corresponding interference effect on the change in peak current of 0.05 μM Zn(II) (**b**). Other experimental and instrumental parameters are as in Fig. 3.

The effects of various ions including Cu^2+^, Pb^2+^, Cd^2+^, Fe^3+^, Na^+^, Ca^2+^, SO_4_^2−^, Cl^−^ and NO_3_^−^ on the current response of Zn(II) was studied. It was performed by adding these interfering ions into a solution containing a known concentration of Zn(II) (0.05 μM which is below the concentration found in the analyzed wastewater sample, 0.34 μM) in different groups with different concentration ratios from 1 to 50 folds at the time of accumulation. The SWASVs in the absence and presence of interfering ions are shown in Fig. 11a and the corresponding changes in peak current are shown in Fig. 11b. From the obtained results (Fig. 11a and b), the presence of Na^+^, Ca^2+^, Cl^−^ and NO_3_^−^ ions in the concentration levels up to 50 fold (group 1); SO_4_^2−^ up to 10 fold (group 2); and Fe^3+^ up to 5 fold (group 3) to the concentration of Zn(II) has only a negligible influence on the current signal of Zn(II). When the Cd^2+^ (group 4), Pb^2+^ (group 5), and Cu^2+^ (group 6) ions were present in equal concentration (each metal ion at once), they have a little effect on the peak current response of Zn(II) (up to approximately 11% current response change). This indicated that even though the oxidation peak potentials of these three ions are quite different/far from the peak potential of Zn(II), the ligand (HDPBA) is able to form complex with thsese metal ions (Cd^2+^, Pb^2+^ and Cu^2+^) besides the Zn(II) ion. In this case, experiments of addition and recovery were performed by the standard addition method and recoveries from 91.70 to 112.3% were obtained. This result reveals that Zn(II) can be reliably determined by the proposed electrode even in the presence of thsese meta ions (Cu^2+^, Cd^2+^, and Pb^2+^) when the standard addition method is used.

### 3.6 The novelty of the study

In this study, a sensitive and selective electrode for the determination of trace levels of Zn(II) was prepared using HDPBA and MWCNTs nanocomposite modified CPE (HDPBA‒MWCNTs/CPE). Cost-effectiveness, simple preparation of the electrode, an easy renewable surface of the developed electrode, and fast analysis are some of the novelties of the present method. The prepared electrode (HDPBA‒MWCNTs/CPE) exhibited high sensitivity and selectivity with excellent reproducibility and stability for the electrochemical detection of Zn(II). The developed electrode has achieved better sensing ability with a wider linear range and a lower detection limit than other electrodes reported in the literature for the detection of Zn(II). The proposed HDPBA‒MWCNTs/CPE was successfully applied in the quantification of Zn(II) in industrial wastewater and tap water samples from different sources.

### 3.7 Real sample analysis

**Fig. 12:**
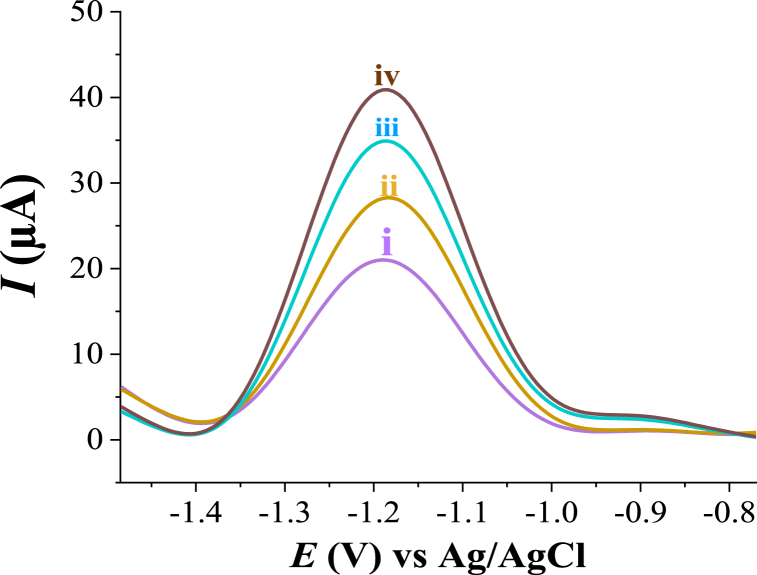
Square wave voltammograms for wastewater with spiking different concentrations of Zn(II): (i) 0.0 μM, (ii) 0.2 μM, (iii) 0.4 μM, and (iv) 0.6 μM. Other experimental and instrumental parameters are as in Fig. 3.

Based on the analytical characteristics of the proposed HDPBA‒MWCNTs/CPE, it was applied for the determination of Zn(II) in real samples in order to evaluate the practical applicability of the method. The real sample analyses were conducted using wastewater and tap water samples that were prepared and pretreated as presented above in the experimental section. The quantification of Zn(II) in water samples was performed by using the spiking method. The results found from the present method were compared and validated with that measured with atomic absorption spectrometry (AAS) method. The obtained results from both methods are given in Table 2. As shown in the table, the original concentration of Zn(II) in the wastewater sample was found to be 0.342 μM. While Zn(II) was not detected in tap water sample (analyte concentrations below the detection limit). The SWASVs for the analyzed wastewater sample are depicted in Fig. 12. Based on the paired *t*-test (at a 95% confidence level, *p* = 0.05), a good agreement was found between the results obtained from the proposed method and a comparative method (AAS). This indicates that the present method is reliable for the quantification of trace concentrations of Zn(II) in real matrixes without significant effects.

**Table 2:** Real sample analysis results (N = 5) of the determination of Zn(II) in water samples using the proposed electrode (HDPBA‒MWCNTs/CPE) and AAS method.

Sample	Added (μM)	Found (μM) (proposed electrode)	Recovery (%)	Found (μM) (AAS method)	Recovery (%)
Wastewater	0	0.342 (±0.014)	–	0.355 (±0.015)	–
	0.200	0.539 (±0.016)	98.50	0.549 (±0.020)	97.00
Tap water	0	ND	–	ND	–
	0.200	0.212 (±0.011)	106.0	0.209 (±0.013)	104.5

ND: not detected.

### 3.8 Electrochemical behavior of N^1^-hydroxy-N^1^,N^2^-diphenylbenzamidine (HDPBA)

#### 3.8.1 Voltammetric behavior of HDPBA in acetonitrile/lithium perchlorate and aqueous solutions

The voltammetric behavior of 0.5 mM of N^1^-hydroxy-N^1^,N^2^-diphenylbenzamidine (HDPBA) in acetonitrile containing lithium perchlorate (LiClO_4_, 0.1 M) and 10 mM tetrabutylammoniumhexafluoro-phosphate (TBAHFP) was studied using GCE. Fig. S2 depicts the cyclic voltammograms (CVs) of the electrolyte (lithium perchlorate) and HDPBA at GCE. As can be seen in Fig. S2a, there are no redox peaks of the electrolyte (LiClO_4_) at GCE. However, HDPBA exhibited both anodic and cathodic peak currents in acetonitrile containing lithium perchlorate (LiClO_4_, 0.1 M) and 10 mM (TBAHFP) using glassy carbon electrode (GCE) (Fig. S2b). The major peak was the anodic peak with a peak potential value at 0.714 V. The anodic peak is due to the oxidation of HDPBA and the cathodic peak might be attributed to the corresponding reduction of HDPBA oxidized form. This shows that the modifier (HDPBA) is redox active in a given supporting electrolyte solution. The effect of concentrations of HDPBA on the voltammetric behavior of the ligand was evaluated by preparing 0.1, 0.5, 1.0, and 1.5 mM of HDPBA in acetonitrile containing LiClO_4_ (0.1 M) and 10 mM TBAHFP solution. Reproducible voltammetric behavior of HDPBA was obtained for all the four concentrations of HDPBA.

Furthermore, the voltammetric characteristics of 7.5% HDPBA incorporated in the CPE were investigated in detail in 0.3 M acetate buffer solution (sodium acetate) of pH 4. The CVs of the unmodified CPE and 7.5% HDPBA-modified CPE are shown in Fig. S3. The unmodified CPE exhibits a small background current of CV in the given potential scan (Fig. S3a). However, the 7.5% HDPBA-modified CPE (Fig. S3b) shows redox peaks (almost at similar peak potential values with that of HDPBA in acetonitrile/LiClO_4_/TBAHFP solution at glassy carbon electrode in acetonitrile solution). It should be noted that the use of two different electrodes (CPE and GCE) in two solutions (acetate solution and acetonitrile/LiClO_4_/TBAHFP solution, respectively) was to investigate whether the two electrodes give significantly different results on the voltammetric behavior of the ligand (HDPBA) or not. No significant difference was obtained in the voltammetric behavior of the HDPBA of the two electrodes.

#### 3.8.2 Effect of pH of buffer solution on the voltammetric behavior of HDPBA at CPE

The effect of buffer solution (ABS) pH on the peak currents and peak potentials of HDPBA-modified CPE was studied. Square wave voltammograms at different pH values of 3–12 were recorded. Well-defined peak currents were obtained in the given range of solution pH with a decrease of peak currents when the pH increased from 3 to 7, then it increases in the pH range of 8–10. The peak current response starts again decreasing at pHs 11 and 12. The peak potential was shifted to the negative direction/value with an increase in the pH of the solution up to pH 9. However, no significant shift in the peak potential was observed at higher pH values (after pH 9). This indicates that the electrode process is determined by protonation reactions.

**Fig. 13:**
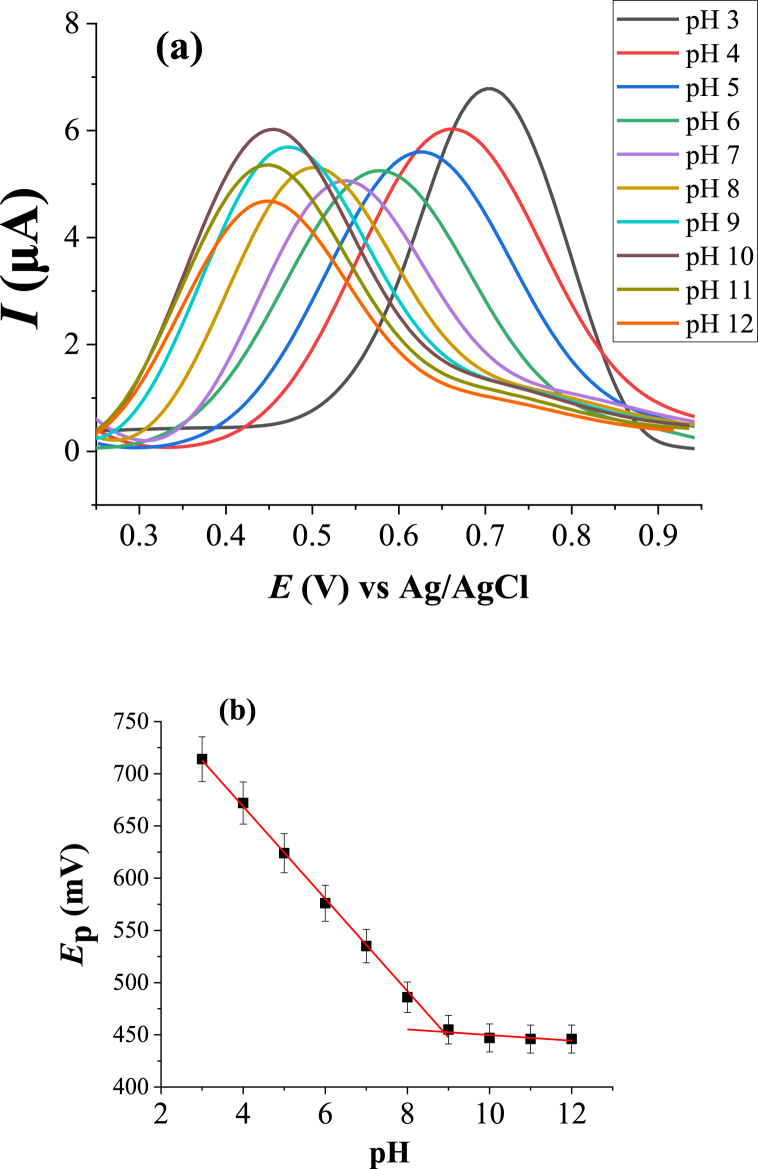
(**a**) Square wave voltammograms of 7.5 wt% HDPBA-modified carbon paste electrode in 0.3 M acetate buffer solution (ABS) at pH 3–12. (**b**) Corresponding anodic peak potentials (E_p_) of HDPBA as a function of pH.

The anodic peak potential shift with variation in the pH was systematically investigated using square wave voltammetry (SWV). The voltammograms of HDPBA in acetate buffer solutions of different pHs and their corresponding peak potentials *(E*_p_) versus pH of buffer solution are depicted in Fig. 13a and b, respectively. As shown in Fig. 13b, the potential–pH curve has two linear parts (pH 3 to 9 and 10 to 12). For the first linear part (pH 3 to 9), a linear correlation was found between the anodic peak potential and pH of a buffer solution with a linear equation and correlation of *E*_p_ (V) = 0.0507(pH) + 0.847, and 0.998, respectively. This shows the linearly dependent of peak potential on the pH. The dependence of the anodic peak potential with a slope was found to be 51 mV/pH. This clearly indicates that the number of protons involved in the electrode process/reaction is similar to the number of electrons, i.e., the ratio of the number of protons to the number of electrons involve in the redox reaction is nearly one [45].

Hence the redox reaction process of the ligand (HDPBA) involves either two protons and two electrons or one proton and one electron. However, it is difficult to give a clear conclusion as to the exact number of electron(s) and proton(s) and also regarding the products of the redox reaction from the results of this study [30,46]. Therefore, we can only conclude that HDPBA electrochemical behavior was found to be highly pH dependent.

## 4 Conclusion

In the present study, N^1^-hydroxy-N^1^,N^2^-diphenylbenzamidine and multi-walled carbon nanotubes composite modified CPE (HDPBA‒MWCNTs/CPE) was prepared for the determination of zinc ions in the B–R buffer solution by the SWASV method. Optimizations of different experimental and instrumental parameters were studied. The advantages of the proposed electrode include simplicity, an easy renewable surface, low cost, good accuracy, and rapid analysis. At optimum experimental conditions, the developed HDPBA‒MWCNTs/CPE showed high sensitivity, good selectivity, and better reproducibility and stability for the determination of Zn(II). In comparison with other electrodes from the literature, the present electrode has better analytical performance with a wider linear range and a very low detection limit than the reported electrodes. The practical usefulness of real sample analysis of the proposed method was investigated by effective quantification of the concentration of Zn(II) in water samples and a comparison of the results using the AAS technique was made. A good agreement is found in the results obtained from the two methods.

Furthermore, the electrochemical behavior of N^1^-hydroxy-N^1^,N^2^-diphenylbenzamidine (HDPBA) was also studied in acetonitrile and aqueous (sodium acetate) solution using GCE and HDPBA incorporate CPE, respectively. The ligand, HDPBA exhibited both anodic and cathodic peak currents in both solutions. The major peak was the anodic peak and whose peak potential value (around 0.714 V) is far from that of Zn(II) (which is at −1.190 V). The redox behavior of HDPBA was found to be dependent on the buffer solution pH.

## Author contribution statement

Endale Tesfaye: Conceived and designed the experiments; Performed the experiments; Analyzed and interpreted the data; Contributed reagents, materials, analysis tools or data; Wrote the paper.

Bhagwan Singh Chandravanshi: Conceived and designed the experiments; Analyzed and interpreted the data; Contributed reagents, materials, analysis tools or data; Wrote the paper.

Negussie Negash, Merid Tessema: Analyzed and interpreted the data; Contributed reagents, materials, analysis tools or data; Wrote the paper.

## Funding

This research was not funded by any funding agency.

## Data availability statement

All the data are included in the manuscript.

## Declaration of competing interest

The authors declare that they have no known competing financial interests or personal relationships that could have appeared to influence the work reported in this paper.
