# Supplementary material for: Electrochemical determination of zinc(II) using N1-hydroxy-N1,N2-diphenylbenzamidine and multi-walled carbon nanotubes modified carbon paste electrode
Source: Heliyon. 2023 Jun 15;9(6):e17346. doi: 10.1016/j.heliyon.2023.e17346 (PMC10293732; doi:10.1016/j.heliyon.2023.e17346)
Supplement: Multimedia component 1 [file mmc1.docx]

**Supplementary Information**

**Figures**

**Fig. S1.** The schematic representation of the proposed complexation mechanism of HDPBA with Zn^2+^.

**Fig. S2.** Cyclic voltammograms of (**a**) 0.10 M lithium perchlorate (LiClO_4_) and (**b**) 0.5 mM HDPBA in acetonitrile containing LiClO_4_ (0.1 M) and 10 mM TBAHFP solution at glassy carbon electrode (GCE). Scan rate: 50 mV/s.

**Fig. S3.** Cyclic voltammograms of (**a**) unmodified CPE and (**b**) 7.5 wt.% HDPBA-modified carbon paste electrode (HDPBA/CPE) in 0.3 M sodium acetate buffer of pH 4.
